# Allopregnanolone alters follicular and luteal dynamics during the estrous cycle

**DOI:** 10.1186/s12958-018-0353-y

**Published:** 2018-04-10

**Authors:** Joana Antonela Asensio, Antonella Rosario Ramona Cáceres, Laura Tatiana Pelegrina, María de los Ángeles Sanhueza, Leopoldina Scotti, Fernanda Parborell, Myriam Raquel Laconi

**Affiliations:** 1 Laboratorio de Fisiopatología Ovárica, Instituto de Medicina y Biología Experimental de Cuyo (IMBECU - CONICET), Mendoza, Argentina; 2 Facultad de Ciencias Veterinarias y Ambientales, Universidad Juan Agustín Maza, Mendoza, Argentina; 3 Laboratorio de Fisiopatología del Ovario, Instituto de Biología y Medicina Experimental (IByME - CONICET), Buenos Aires, Argentina; 4 Facultad de Ciencias Médicas, Universidad de Mendoza, Mendoza, Argentina

**Keywords:** Allopregnanolone, Folliculogenesis, Angiogenesis, Apoptosis, Steroidogenesis, *Corpora lutea*, Luteolysis

## Abstract

**Background:**

Allopregnanolone is a neurosteroid synthesized in the central nervous system independently of steroidogenic glands; it influences sexual behavior and anxiety. The aim of this work is to evaluate the indirect effect of a single pharmacological dose of allopregnanolone on important processes related to normal ovarian function, such as folliculogenesis, angiogenesis and luteolysis, and to study the corresponding changes in endocrine profile and enzymatic activity over 4 days of the rat estrous cycle. We test the hypothesis that allopregnanolone may trigger hypothalamus - hypophysis - ovarian axis dysregulation and cause ovarian failure which affects the next estrous cycle stages.

**Methods:**

Allopregnanolone was injected during the proestrous morning and then, the animals were sacrificed at each stage of the estrous cycle. Ovarian sections were processed to determine the number and diameter of different ovarian structures. Cleaved caspase 3, proliferating cell nuclear antigen, α-actin and Von Willebrand factor expressions were evaluated by immunohistochemistry. Luteinizing hormone, prolactin, estrogen and progesterone serum levels were measured by radioimmunoassay. The enzymatic activities of 3β-hydroxysteroid dehydrogenase, 3α-hydroxysteroid oxidoreductase and 20α-hydroxysteroid dehydrogenase were determined by spectrophotometric assays. Two-way ANOVA followed by Bonferroni was performed to determine statistical differences between control and treated groups along the four stages of the cycle.

**Results:**

The results indicate that allopregnanolone allopregnanolone decreased the number of developing follicles, while atretic follicles and cysts increased with no effects on normal cyclicity. Some cysts in treated ovaries showed morphological characteristics similar to luteinized unruptured follicles. The apoptosis/proliferation balance increased in follicles from treated rats. The endocrine profile was altered at different stages of the estrous cycle of treated rats. The angiogenic markers expression increased in treated ovaries. As regards *corpora lutea*, the apoptosis/proliferation balance and 20α-hydroxysteroid dehydrogenase enzymatic activity decreased significantly. Progesterone levels and 3β-hydroxysteroid dehydrogenase enzymatic activity increased in treated rats. These data suggest that allopregnanolone interferes with steroidogenesis and folliculogenesis at different stages of the cycle.

**Conclusion:**

Allopregnanolone interferes with *corpora lutea* regression, which might indicate that this neurosteroid exerts a protective role over the luteal cells and prevents them from luteolysis. Allopregnanolone plays an important role in the ovarian pathophysiology.

## Background

The brain has always been considered a target for sex steroid hormones produced by peripheral steroidogenic organs (gonads and the adrenal glands); it is now well accepted that the brain synthesizes neurosteroids *de novo*, and converts circulating steroids to neuroactive steroids [1]. Regardless of their origin, steroids affect brain function through actions at their cognate receptors, or by affecting receptors whose primary transmitter is not a steroid (e.g., GABA receptors). Allopregnanolone (3α-hydroxy-5α-pregnan-20-one, ALLO) is a neurosteroid synthesized in the brain, adrenal glands and gonads from progesterone (PG) metabolism by the action of the 3α-hydroxysteroid dehydrogenase (3α-HSD) enzyme [2]. It can also be produced *de novo* from cholesterol in the brain [3] and it acts as a potent neurosteroid that can alter membrane excitability [4, 5].

One of the most intriguing questions has been the relationship of peripheral steroids with neurosteroids. Free steroids (i.e., steroids not bound to carrier proteins) are capable of diffusing across the blood–brain barrier to bind both membrane-associated steroid receptors and intracellular receptors. Thus, levels of a particular steroid in the brain are a composite of steroids from the periphery, converted peripheral steroids, and neurosteroids. Additionally, hormonal steroids also regulate the site-specific synthesis of neurosteroid levels [6, 7] and their cognate receptors [8–10] that affect neurosteroid levels and function. ALLO is a positive allosteric modulator of GABA_A_ receptors; in fact, ALLO enhances GABA activity at nanomolar concentrations and opens chloride channels at micromolar concentrations [5, 11–13]. Production of ALLO increases in response to stress in order to decrease pain sensitivity and restore physiologic homeostasis [14–16]. In previous works from our laboratory, ALLO reduced anxiety and inhibited lordosis, and thus it influences sexual behavior in the female rat [17, 18]. Several studies revealed that higher than physiological ALLO concentrations inhibit GnRH and sexual behavior in rats [18–22]. These findings prompted us to consider ALLO not only as a modulator of hypothalamic function but also of ovarian physiology.

Follicular development process, ovulation, *corpora lutea* (CL) formation and luteolysis are critical events that occur cyclically in the rat ovary, which are subject to a complex endocrine regulation. In previous studies, we reported that ALLO injected via intracerebroventricular (i.c.v.) during proestrous (PE) morning inhibited luteinizing hormone (LH) surge and, consequently, decreased the number of ovulated oocytes in estrous (E) rats. ALLO also increased prolactin (PRL) and PG serum levels, and decreased the number of apoptotic *nuclei* in luteal cells [22]. Furthermore, ALLO interfered with folliculogenesis process since the number of developing follicles and new CL were reduced in E treated rats. ALLO also increased the number of cystic structures and altered steroidogenesis at E stage; however, normal cyclicity was not altered [23]. While the role of ALLO is well documented in the central nervous system (CNS), the functions on the ovarian physiology remains to be fully explored.

As we found previously, ALLO affected several important ovarian parameters that might impair reproduction [22, 23]; therefore, we aim to investigate if these alterations remain throughout the next estrous cycle. We evaluated the effect of a single pharmacological dose of ALLO, injected in the PE morning, on (a) number of ovarian structures, (b) follicle and CL sizes, (c) apoptosis, proliferation and angiogenesis processes (d) LH, PRL, 17β-estradiol (E2) and PG serum levels, and (e) 3β-hydroxysteroid dehydrogenase (3β-HSD), 3α-hydroxysteroid oxidoreductase (3α-HSOR) and 20α-hydroxysteroid dehydrogenase (20α-HSD) enzymatic activities. The effects of ALLO on these parameters were evaluated on E, diestrous 1 (D1), diestrous 2 (D2) and PE stages of the cycle. The relevance of this study is that it is the first evidence of the effect of ALLO on the whole cycle and its putative pathological consequences.

## Methods

### Animals

Adult female Sprague Dawley rats (*Rattus norvegicus*) of 200-250 g were maintained under controlled conditions of temperature and light (12 h light/12 h darkness photoperiod). They were housed in groups of four animals *per* cage with food (standard rat chow Cargil, Córdoba, Argentina) and water available *ad libitum*. Vaginal smears from each rat were observed daily (07:00-09:00 am) with a light microscope (Zeiss, Germany) to determine the stage of the estrous cycle. Only those animals exhibiting two or more consecutive 4 or 5-day cycle were used. All protocols were previously approved by the Institutional Committee for Care and Use of Experimental Animals (CICUAL N° 141021) and conducted according to the National Institutes of Health Guide for the Care and Use of Laboratory Animals of the National Research Council (National Academies, U.S.A., 8th Edition, 2011).

### Drugs

Allopregnanolone (Sigma Chemical Co., St. Louis, MO, USA), Penicillin G Benzathine (Riched, Argentina), Ketamine HCL (Hollliday - Scott S.A, Buenos Aires, Argentina) and Xylazine (Koning Laboratories, Buenos Aires, Argentina) were used for experimental and surgical procedures. Stocks of ALLO were initially dissolved in propylene-glycol to a concentration of 0.6 mM. The dose of ALLO used in the experiments (6 μM) was obtained by dilutions in Krebs Ringer bicarbonate glucose buffer (KREBS) at pH 7.4. Bouin solution (Biopur Diagnostics, Santa Fe, Argentina) and Canada Balsam Synthetic (Biopack, Buenos Aires, Argentina) were purchased for histological procedures. Anti-cleaved caspase 3 (CASP3) (Biocare Medical, #CP229C, California, USA) raised in rabbit, anti-proliferating cell nuclear antigen (PCNA) raised in rabbit (Santa Cruz Biotechnology, sc-7907, USA), anti-α-actin raised in mouse (Santa Cruz Biotechnology, sc-56499, USA), polyclonal anti-Von Willebrand factor raised in rabbit (Dako Cytomation, A0082, USA), Anti-Rabbit HRP IgG (Sigma Aldrich, A4914, USA), anti-mouse HRP IgG (R&D Systems HAF007, Minnesota, USA), and avidin-biotinylated HRP complex (Vectastain ABC system; Vector Laboratories, Burlingame, CA, USA) were used for immunohistochemistry technique.

### Surgical Procedures

A stainless steel cannula was stereotaxically inserted into the right lateral ventricle under Ketamine/Xylazine anesthesia (50 mg/kg and 5 mg/kg body weight, respectively). The following coordinates were used: AP +0.4 mm, L +1.5 mm, and DV -4 mm [24]. At the end of the surgery, the cannula was sealed with a stainless steel wire to avoid obstruction. In order to prevent infections, each animal received a subcutaneous injection of 0.2 ml of 1.200.000 UI penicillin G Benzathine (1UI = 0.6 μg; 72 mg/rat). After the surgery, the animals were housed singly in plexiglas cages and maintained for a week undisturbed in order to recover the estrous cycle. At the end of the experiments, after decapitation, the location of the guide cannula was confirmed by the injection of blue ink. Only animals with confirmed microinjection into right lateral ventricle were included in the experiments.

### Experimental design

**Fig. 1:**
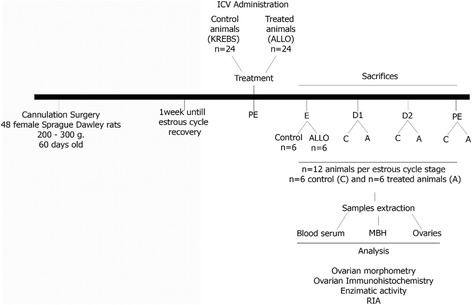
Experimental design of the study. A: ALLO treated group, C: control, E: estrous, ICV: intracerebroventricular, D1: diestrous 1, D2: diestrous 2, MBH: medial basal hypothalamus, PE: proestrous, RIA: radioimmunoassay

The rats were randomly assigned to either control or ALLO treated groups for each estrous cycle stage (n = 6/group, total of 48 animals) (Fig. 1). On the PE morning, the experimental groups received a single i.c.v. injection of ALLO: 6 μM, 1 μl, during 60 seconds to avoid reflux. A pharmacological dose of ALLO was administered as used in our previous reports of anxiety, memory and sexual behavior [17, 18, 21–23]. Control animals were injected with KREBS solution, containing propylene-glycol in equivalent concentrations to the used in ALLO groups. Six animals for each day of collection for experimental group were euthanized by decapitation 24 h (E), 48 h (D1), 72 h (D2) and 96 h (PE) after a unique injection of KREBS or ALLO. Serum samples were collected from each rat after blood centrifugation and stored at -20°C for RIA. From each rat, the right ovary and medial basal hypothalamus (MBH) were removed for 3β-HSD, 3α-HSOR and 20α-HSD enzymatic activity measurements. The left ovary was preserved for histological assays. One set of ovarian slides was stained with hematoxylin-eosin to quantify the number of structures at different stages of follicular development, and the other sections were used for immunohistochemistry.

### Ovarian morphometry

The left ovaries from each rat were removed and immediately fixed in Bouin solution for 12 h. Then, dehydrated in a series of increasing concentration alcohols and finally embedded in paraffin. Paraffin sections of 5 μm were taken every 50 μm and mounted onto microscope slides to prevent counting the same structure twice, according to the method described by Woodruff *et al.* (1990) [25]. Follicles were classified as primary (PF; a single layer of cuboidal granulosa cells, preantral (PAF; more than one layer of granulosa cells), early antral (EAF; three or more granulosa layers with an incipient antrum), antral (AF; more than three granulosa layers and a clearly defined antral space) and Graafian follicles (GF; polarized oocyte, defined cumulus granulosa layer and a cavity occupying most of the follicular volume) [26, 27]. *Corpora lutea* (CLs) were identified by the presence of large luteal cells and their characteristic cytoplasmic eosinophilia, surrounded by small lutein cells [28]. *Corpora albicans* were scarce, and they were not taken into account. Morphological characteristics of atretic follicles (ATF) include pyknotic *nuclei* (cell degeneration), detachment of the granulosa cell layer from the basal *laminae*, hypertrophy of external theca and oocyte degeneration [29]. Cysts can be subdivided into follicular and luteal cysts: follicular cysts are surrounded by a thin granulosa layer (≤ 3 mm) and contain a large antral cavity (the oocyte may be absent or present), while luteinized unruptured follicles (LUF) consist of an oocyte surrounded by luteinized granulosa cells and have a thicker wall (> 3 mm) [30–32]. The number of ovarian structures was determined in three ovarian sections from each ovary (three sections *per* ovary; n=6). Numbers of PF, PAF, EAF, AF, GF, CL, ATF and cysts (including LUFs) were determined for each left ovary/animal in each experimental group; then they were expressed as Mean ± S.E.M. of the number of structures in each ovary at each stage of the cycle.

The diameters of EAF, AF, GF and CL were measured using Image J software (Image Processing and Analysis in Java; National Institutes of Health, Bethesda, MD, USA). Results were expressed as mean diameter ± S.E.M.

### Immunohistochemistry

Ovarian tissue sections were processed for *in situ* localization of CASP3, PCNA, α-actin and von Willebrand factor. Ovarian sections were deparaffinized in xylene and rehydrated in descending graduation ethanol washes. Endogenous peroxidase activity was blocked with 3% hydrogen peroxide in PBS and nonspecific binding was blocked with 2% BSA for 20 min at room temperature. Sections were incubated with the primary antibodies: CASP3 (1:300), PCNA (1:300), α-actin (1:100) and von Willebrand (1:100) overnight at 4°C in a humid chamber. After PBS washing, the slides were incubated with the corresponding secondary antibodies (anti-rabbit HRP-IgG 1:1000, anti-mouse HRP-IgG 1:1000) for 2 h and then with avidin-biotinylated HRP complex (1:400) for 30 min. Protein expression was visualized using diaminobenzidine staining. The negative controls were obtained in the absence of primary antibodies. The reaction was stopped using distilled water, counterstained with hematoxylin and dehydrated before mounting. The images were digitized using a camera (Nikon, Melville, NY, USA) mounted on a conventional light microscope (Nikon).

Apoptosis and cellular proliferation were evaluated in EAF, AF, GF and CL by CASP3 and PCNA immunohistochemistry, respectively. EAF, AF and GF are the most sensitive ovarian structures to degeneration by atresia, since they have follicle-stimulating hormone (FSH) and LH receptors and are capable of sensing the balance between survival/apoptotic factors [33, 34]. Six ovaries from six different rats were used *per* experimental group (n=6, 48 ovaries in total). From each ovary, four follicles and four CL were selected and numbered to be analyzed. Within each follicle and each CL, four randomly selected fields were identified and numbered. In each ovary, thirty-two fields were analyzed in total: 16 fields for follicles and 16 fields for CL. Cell counting was performed using Image J software (NIH, Bethesda, MD). Proliferation or apoptotic index was calculated for each selected field as follows: CASP 3 or PCNA-positive cells divided by the total number of cells in the whole field. Results were expressed as mean ± S.E.M of positive immunostained cells for CASP3 or PCNA on the base of this raw data.

ALLO effects on ovarian angiogenesis were evaluated by immunohistochemistry for α-actin and von Willebrand at each stage of the estrous cycle. Six randomly selected fields were analyzed from each ovarian section (six sections *per* ovary; n=6). The percentage of endothelial cell area positively marked, either for α-actin or von Willebrand, with respect to the total area of the ovary was quantified using Image J software. The results obtained were expressed as mean ± S.E.M. of endothelial cell area positively marked.

### RIA for LH, PRL, E2 and PG

Trunk blood was collected from each experimental group (n=6) in heparinized tubes and centrifuged during 15 min at 1500g (Beckman TJ-6RS). The plasma obtained was kept frozen (-20°C) until hormone assays were run. LH and PRL serum levels were determined by RIA using kits supplied by the National Hormone Pituitary Program, USA. The LH standard was NIDDK-rLH-RP-3 and the antibody NIDDK rLH-S-11. The PRL standard was NIDDK-rPRL-RP-3 and the antibody NIDDK-rPRL-S-9. The sensitivity of the assay was 0.5 ng/tube. The intra- and inter-assay coefficients of variation were 9 and 11%, respectively. The data were expressed in nanograms per milliliter (ng/ml) of serum in terms of NIDDK-rLH-RP-1 and NIDK-rPRL-RP-3 reference preparation. E2 serum concentration was determined by RIA using a commercial kit (Radim, Pomezia, Italy) based on competition between antigens labeled with iodine 125 (radioactive conjugate) and non-labeled antigens (calibrator sample) for specific binding sites in antiserum-coated tubes [22]. PG serum concentration was measured using a commercially obtained kit (Diagnostic Products Corporation, LA, CA, USA). The sensitivity of the assay was 0.02 ng/ml, and the inter- and intra-assay coefficients of variation were 5% and 6%, respectively. For each hormone analyzed, two measurements *per* animal *per* experimental group were obtained, and then the average between them was calculated. This final value was used to obtain mean ± S.E.M. for statistical analysis of LH, PRL, E2 and PG *per* experimental group.

### Spectrophotometric measurements of enzymatic activity

The MBH and the ovaries were homogenized with a glass homogenizer at 0° C in a solution containing: 0.7 ml of 0.1 M Tris-HCl + 1 mM EDTA buffer (pH 8). The activities of 3β-HSD, 3α-HSOR and 20α-HSD were measured as described previously with minor modifications [21, 35, 36]. Lowry method was used for protein determination using bovine serum albumin (BSA) as standard. The homogenates were centrifuged at 105000g for 60 min, using a Beckman L T40.2 ultracentrifuge. The supernatants were used for determination of 20α-HSD activity. The precipitates were re-homogenized with 0.25 M sucrose and then centrifuged at 1200g for 5 min. The supernatants obtained were used as the enzyme solution to determine 3β-HSD activity. Then pregnenolone, the substrate for the reaction of 3α-HSOR, was added to the reaction mix. The latter contained Glycine-NaOH (pH 9.4), BSA, NAD+ and a fraction of the enzyme solution. The enzymatic activities were determined spectrophotometrically using a Zeltec spectrophometer. The assay of each enzyme measured the reduction of NAD+ or the oxidation rate of NADPH cofactor at 340 nm, respectively, as an increase in absorbance in 1 min at 37°C [35, 37, 38]. A fraction of the enzymatic solution was reserved for protein quantification. The values of enzymatic activity were expressed as mUI/mg protein/min.

### Statistical analysis

Raw data obtained were analyzed using Graph Pad Prism version 5.03 for Windows (Graph Pad Software, California, USA). Results were expressed as mean ± S.E.M. The aim of this work is to determine how certain parameters of the ovarian physiology are affected by two variables: treatment and stage of the estrous cycle. Therefore, ANOVA two-way was performed in all the experiments, followed by Bonferroni’s posttest to determine the individual differences between control and ALLO groups at each stage of the cycle. D’ Agostino-Pearson normality test was used prior to ANOVA two-way. Differences were considered significant at *p<*0.05.

## Results

### Effect of ALLO on ovarian morphometry

Our results on E stage were published previously [22, 23] and they were included in this article in order to make comparisons easier. The results of the morphometric analysis are showed in Table 1. The number of PF decreased in D2 treated rats (*p<*0.01). No significant differences were found in the number of PAF at any stage of the estrous cycle between control and ALLO groups. The number of EAF diminished significantly in treated rats at all stages of the cycle (E *p*<0.01, D1 *p*<0.001, D2 *p*<0.001 and PE *p*<0.05). AF decreased in treated rats of E (*p*<0.05), D1 (*p*<0.001) and D2 (*p*<0.01). GF number decreased in ALLO treated rats at E stage (*p*<0.001). There was a decrease in the number of CL in D2 (*p*<0.01), but there were no significant differences in E, D1 and PE stages. ATF number increased substantially in ALLO treated ovaries from all stages of the estrous cycle (E *p*<0.001, D1 *p*<0.01, D2 *p*<0.001 and PE *p*<0.01). The number of cysts increased in E (*p*<0.001), D1 (*p*<0.05) and D2 (*p*<0.05) treated groups; no cysts were found at PE stage, neither in control nor in ALLO groups. Finally, we found a decrease in EAF diameter at E and a decrease in CL diameter (*p*<0.01) at D1 stage treated groups.

**Table 1:** Number and diameter of different ovarian structures in control and ALLO treated rats along the estrous cycle

Group (cyclic stage)	Control (E)	ALLO (E)	Control (D1)	ALLO (D1)	Control (D2)	ALLO (D2)	Control (PE)	ALLO (PE)
PF (n)	3.17 ± 0.75	2.83 ± 0.75	6.50 ± 0.65	5.75 ± 0.75	6.80 ± 1.74	5.00 ± 0.77**	3.50 ± 0.43	2.60 ± 0.40
PAF (n)	3.94 ± 0.56	2.77 ± 0.33	6.25 ± 1.32	7.75 ± 1.44	8.25 ± 1.32	7.40 ± 2.48	3.17 ± 0.65	1.80 ± 0.80
EAF (n)	4.83 ± 0.31	2.83 ± 0.31**	5.25 ± 0.85	2.50 ± 0.50***	9.20 ± 1.74	1.75 ± 2.08***	2.50 ± 0.34	0.80 ± 0.49*
AF (n)	2.67 ± 0.33	1.33 ± 0.33*	3.50 ± 0.65	0.50 ± 0.50***	4.40 ± 1.03	2.60 ± 1.08**	1.50 ± 0.56	1.00 ± 0.45
GF (n)	2.83 ± 0.31	0.67 ± 0.21***	0.75 ± 0.48	0.5 ± 0.5	0.08 ± 0.02	0.80 ± 0.58	0.67 ± 0.49	0.2 ± 0.2
CL (n)	7.65 ± 2.20	6.35 ± 1.10	7.25 ± 0.85	8.25 ± 1.18	13.50 ± 0.50	9.60 ± 0.93**	6.16 ± 1.08	8.40 ± 1.33
ATF (n)	2.75 ± 1.10	4.77 ± 0.90***	7.67 ± 1.20	11.75 ± 1.03**	5.00 ± 0.71	12.80±2.25***	9.33 ± 0.61	12.20 ± 0.97**
Cysts (n)	2.00 ± 0.55	6.00 ± 1.33***	0.01 ± 0.01	0.63 ± 0.26*	0.01 ± 0.01	0.63 ± 0.26*	0	0
EAF diameter (μm)	244.9 ± 19.84	208.70±14.14*	191.00 ± 21.48	189.30 ± 19.17	220.50 ± 31.65	212.80 ± 23.41	216.50 ± 25.55	234.30 ± 5.22
AF diameter (μm)	399.6 ± 26.25	413.15 ± 30.30	415.10 ± 37.51	420.80 ± 49.45	355.50 ± 37.82	378.70 ± 70.96	402.90 ± 12.97	375.00 ± 20.02
GF diameter (μm)	650.9 ± 34.03	603.62 ± 65.12	639.50 ± 20.01	646.10 ± 1.37	654.30 ± 55.29	614.30 ± 29.70	670.76 ± 32.98	689.87 ± 33.98
CL diameter (μm)	758 ± 65.01	836.30 ± 82.30	1019 ± 137.70	848 ±82.32**	946.60 ± 99.56	882.10 ± 97.88	1006 ± 66.93	999.89 ± 61.55

Number of ovarian structures (n) are expressed as Mean ± S.E.M. of primary (PF), preantral (PAF), early antral (EAF), antral (AF), Graafian (GF), atretic follicles (ATF), *corpora lutea* (CL) and cysts (follicular and luteal cysts). Mean diameter (μm) ± S.E.M of EAF, AF, GF and CL along the estrous cycle from control and ALLO treated rats. Two-way ANOVA followed by Bonferroni’s *post hoc*, (n=6), ns *p*>0.05, *p<0.05, **p<0.01, ***p<0.001.

### Effect of ALLO on ovarian apoptosis, proliferation and angiogenesis

**Fig. 2:**
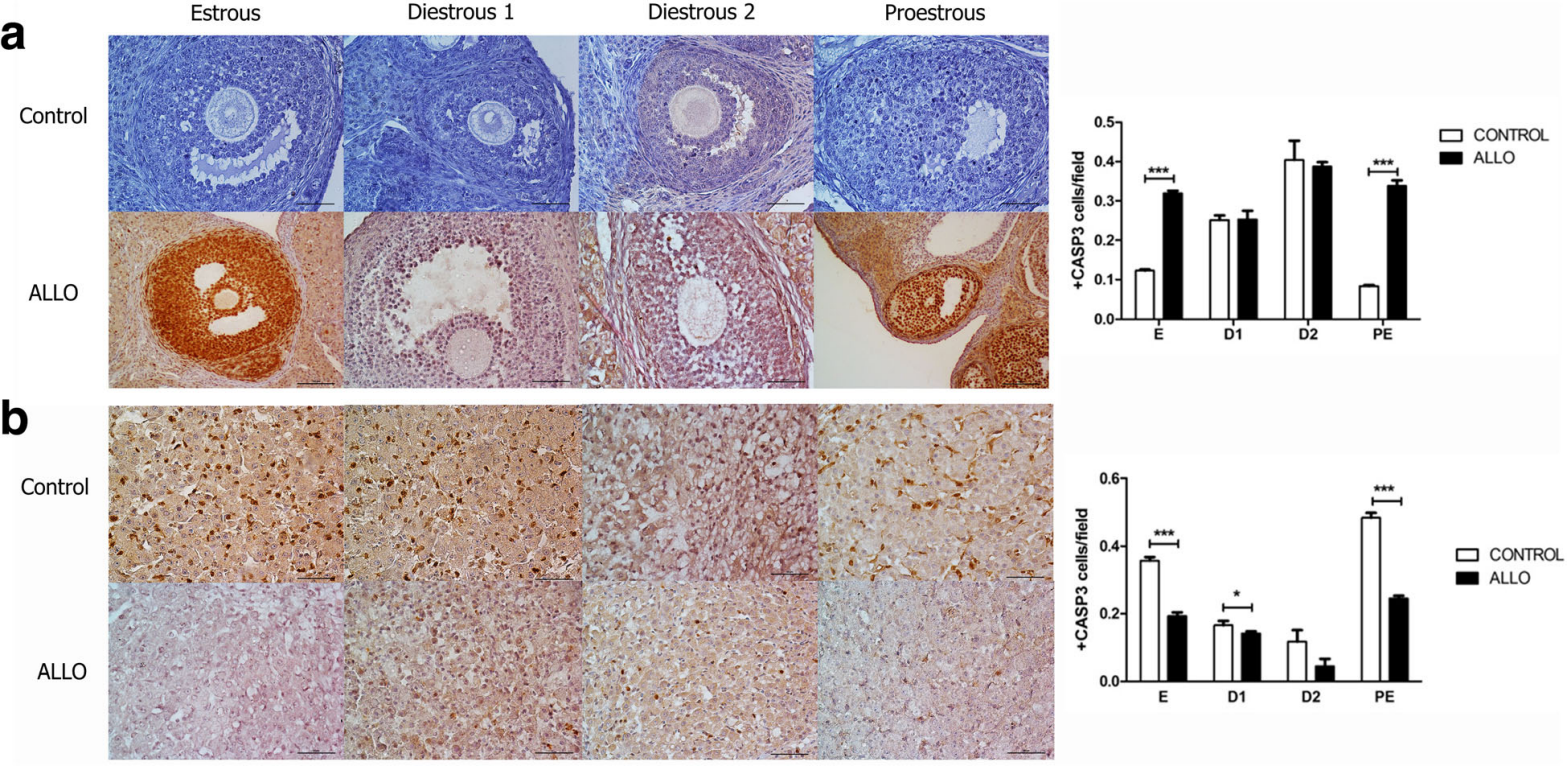
Effect of ALLO on the apoptotic index (cleaved CASP3 positive immunostained cells over the total number of cells) in follicles (**a**) and *corpora lutea* (**b**). Control groups follicles (1A upper panel), ALLO treated follicles (1A lower panel), control groups CL (1B upper panel), ALLO treated groups CL (1B lower panel); E, D1, D2, PE from left to right. Representative photomicrographs of follicles and *corpora lutea* immunostained for cleaved CASP3 at each stage of the estrous cycle. Values are expressed as mean ± S.E.M. Two-way ANOVA followed by Bonferroni’s post hoc, (*n*=6), *p<0.05, ***p<0.001. Magnification: follicles 200x, CL 400x

Cleaved CASP3 expression was evaluated by immunohistochemistry in EAF, GF and CL from control and ALLO groups along the estrous cycle (Fig. 2). Cleaved CASP3 immunoreactivity was increased in granulosa cells at E and PE in ALLO treated groups, compared to control groups (*p*<0.001). No differences were observed in the apoptotic index in follicles neither in D1 nor D2 stages between control and ALLO groups (Fig. 2a). In CL, cleaved CASP3 expression decreased in E, D1 and PE ALLO groups (*p*<0.001, *p*<0.05 and *p*<0.001, respectively) (Fig. 2b).

**Fig. 3:**
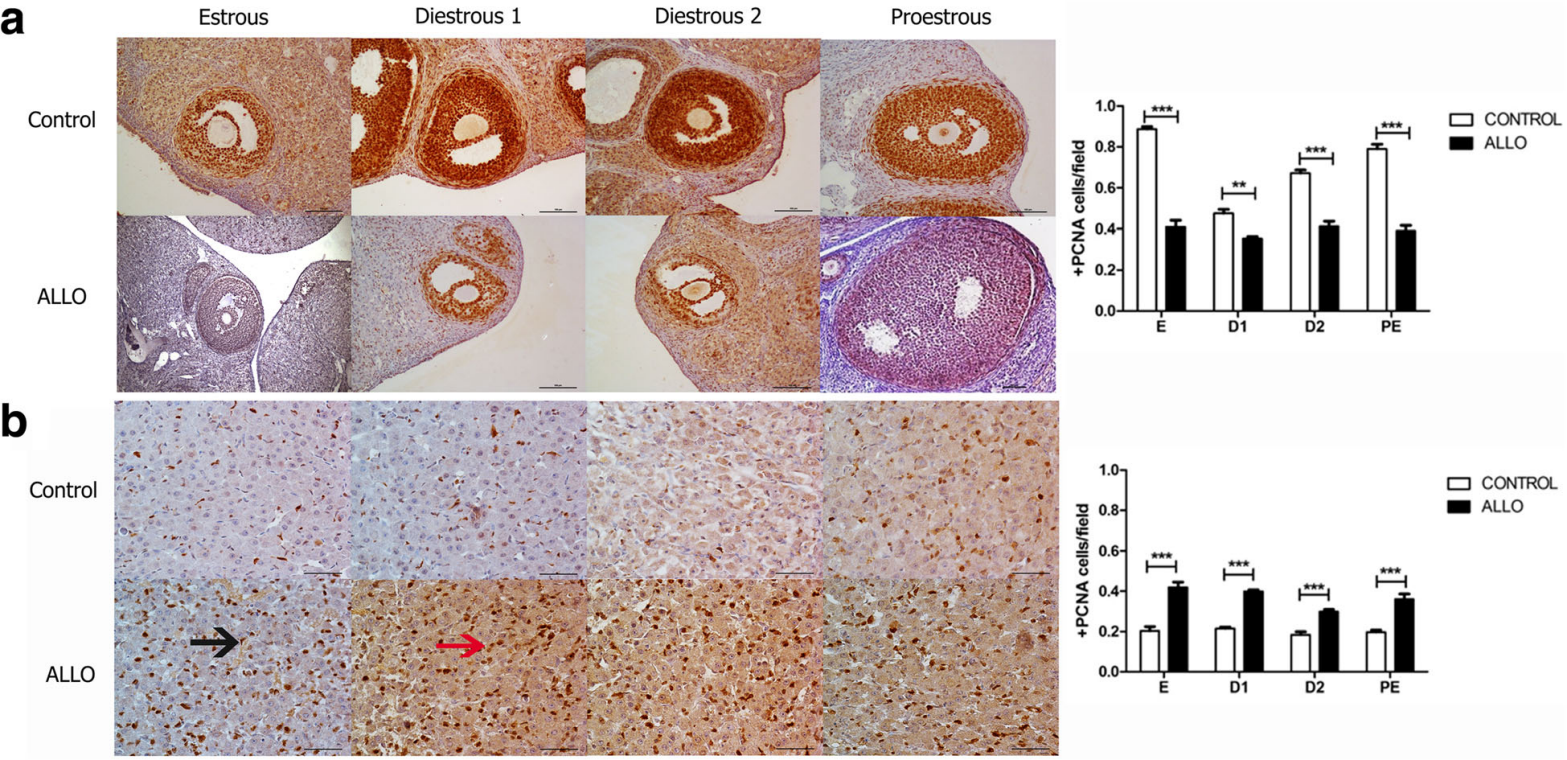
Effect of ALLO on the proliferation index (PCNA positive immunostained cells over the total number of cells) in follicles (**a**) and *corpora lutea* (**b**). Control groups follicles (**a** upper panel), ALLO treated follicles (**a** lower panel), control groups CL (**b** upper panel), ALLO treated groups CL (**b** lower panel); E, D1, D2, PE from left to right. Representative photomicrographs of follicles and *corpora lutea* immunostained for PCNA at each stage of the estrous cycle. Black arrow indicate proliferating endothelial cells, red arrow indicate proliferating small luteal cells. Values are expressed as mean ± S.E.M. Two-way ANOVA followed by Bonferroni’s post hoc, (*n*=6), *p<0.05, ***p<0.001. Magnification: follicles 200x, CL 400x

PCNA immunoreactivity in granulosa cells decreased at all stages of the estrous cycle in ALLO treated groups, in comparison to control ones (E *p*<0.001, D1 *p*<0.01, D2 *p*<0.001 and PE *p*<0.001; Fig. 3a). On the contrary, PCNA immunoreactivity in CL increased in ALLO treated groups at all stages of the estrous cycle (*p*<0.001; Fig. 3b).

**Fig. 4:**
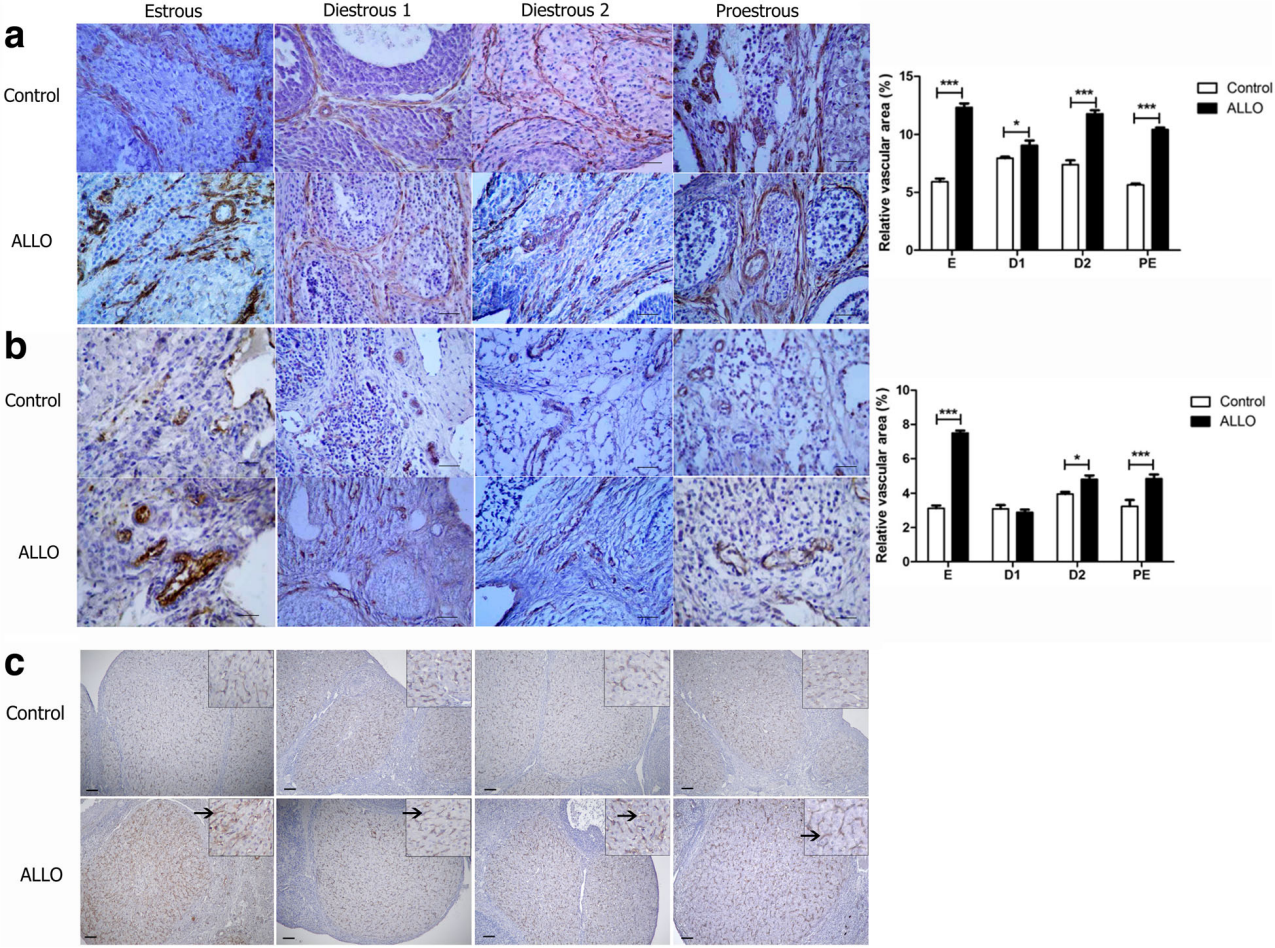
Effect of ALLO on ovarian relative vascular area. Representative photomicrographs of ovaries immunostained for α-actin (**a**) and von Willebrand factor (**b**) at each stage of the estrous cycle. α-actin control groups (**a** upper panel), α-actin ALLO treated groups (**a** lower panel); von Willebrand factor control groups (**b** upper panel), von Willebrand factor ALLO treated groups (**b** lower panel). Values are expressed as mean ± S.E.M of the relative vascular area. Two-way ANOVA followed by Bonferroni’s post hoc (*n*=6), *p<0.05, ***p<0.001. Bar = 50 μm. (**c**) Representative photomicrographs of von Willebrand factor immunostaining in *corpora lutea* of control (**c**, upper panel) and ALLO treated animals (**c**, lower panel). Inset shows higher magnification images (40x). Arrows indicate von Willebrand factor staining of endothelial cells. Bar = 50 μm

In order to evaluate the effect of ALLO on endothelial cell density and stability, histological ovarian slides were immunostained against α-actin and von Willebrand angiogenic factors. ALLO increased the vascular area labeled with α-actin at all stages of estrous cycle (E *p*<0.001, D1 *p*<0.05, D2 *p*<0.001 and PE *p*<0.001; Fig. 4). Similarly, ALLO augmented the vascular area labeled with von Willebrand factor at E (*p*<0.001), D2 (*p*<0.05) and PE (*p*<0.001) stages, with no significant differences at D1 (Fig. 4). Fig. 4c shows that cells immunostained with von Willebrand factor in CL correspond to endothelial cells.

### Effect of ALLO on LH, PRL, E2 and PG serum levels

**Fig. 5:**
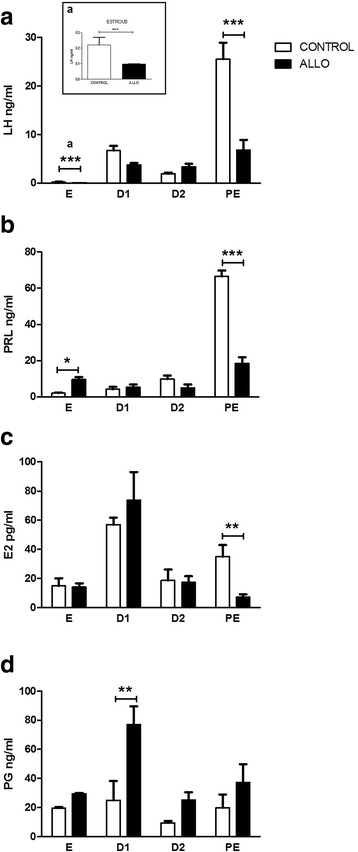
LH (**a**), PRL (**b**), E2 (**c**) and PG (**d**) serum levels from control (white bars) and ALLO treated (black bars) groups along the estrous cycle. E: estrous, D1: diestrous 1, D2: diestrous 2, PE: proestrous. Values correspond to mean ± S.E.M. of hormones serum levels *per* experimental group. Two-way ANOVA followed by Bonferroni’s *post hoc* (n=6), *p<0.05, **p<0.01 and ***p<0.001

Hormone serum levels from control and treated groups along the estrous cycle are presented in Fig. 5. ALLO injection induced a significant decrease in LH serum levels at E (control 0.22 ± 0.05 ng/ml vs. ALLO 0.098 ± 0.001 ng/ml, *p*<0.001) and PE stages (control 25.57 ± 3.37 ng/ml vs. ALLO 6.84 ± 2.07 ng/ml, *p*<0.001) (Fig. 5a, inset a). As regards PRL serum levels, ALLO induced an increase at E (control 2.10 ± 0.30 ng/ml vs. ALLO 9.60 ± 1.20 ng/ml, *p*<0.05) and a decrease at PE stage (control 65.56 ± 3.21 ng/ml vs. ALLO 18.37 ± 3.60 ng/ml, *p*<0.001) (Fig. 5b). E2 serum concentration decreased significantly in ALLO treated rats at PE (control 34.90 ± 8.09 pg/ml vs. ALLO 7.12 ± 2.01 pg/ml, *p*<0.01) (Fig. 5c). PG serum levels increased at D1 in ALLO treated rats (control 25.02 ± 13.22 ng/ml vs. ALLO 77 ± 12.44 ng/ml, *p*<0.01) (Fig. 5d).

### Effect of ALLO on the enzymatic activities of 3β-HSD, 3α-HSOR and 20α-HSD in MBH and ovaries

**Fig. 6:**
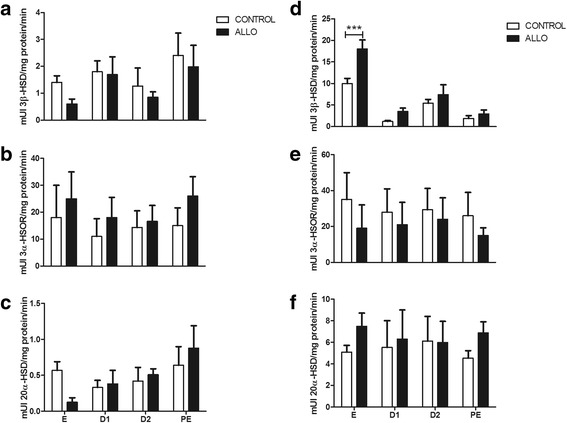
Enzymatic activities of 3β-HSD (**a**–**d**), 3α-HSOR (**b**–**e**) and 20α-HSD (**c**–**f**) in the medial basal hypothalamus (MBH, left panel) and in the ovaries (right panel) at each stage of the estrous cycle. **e**: estrous, D1: diestrous 1, D2: diestrous 2, PE: proestrous. White bars correspond to control groups and black bars to ALLO treated groups. Values correspond to mean ± S.E.M. Two-way ANOVA followed by Bonferroni’s *post hoc* (*n*=6), ***p<0.001

3β-HSD enzymatic activity increased in treated ovaries at E (control 10 ± 1.2 mUI 3β-HSD/mg protein/min vs. ALLO 18 ± 2.1 mUI 3β-HSD/mg protein/min, *p*<0.001) (Fig. 6d). There was no effect of ALLO on 3α-HSOR (Fig. 6b and e) and 20α-HSD (Fig. 6c and f) enzymatic activity, neither in MBH nor in the ovaries.

## Discussion

Allopregnanolone is the most active PG metabolite, and it has multiple functions, in addition to its role as neuromodulator in the nervous system. ALLO is also involved in the regulation of ovarian physiology [22, 23]. However, the concrete correlation of ALLO with follicles and *corpora lutea* dynamics is still not well understood.

The data presented in this study showed, for the first time, how a single pharmacological dose of ALLO can alter the physiological dynamics of follicles and *corpora lutea* during the estrous cycle. Our research was conducted from different approaches: morphology of ovarian structures, molecular physiology (apoptosis, proliferation and angiogenesis) and steroidogenic process (hormone levels and enzymatic activity profiles).

In this work, the injection of ALLO decreased the number of EAF, AF and GF and increased ATF at several stages of the cycle; then, the processes of follicle maturation and selection have been impaired. On the other hand, the number of apoptotic granulosa cells increased and proliferating granulosa cells decreased at all stages in ALLO treated groups. These results suggest that ALLO promotes atresia of developing follicles by stimulating apoptosis on granulosa cells, and thus alters the apoptosis/proliferation balance in the whole follicle.

Granulosa cells are the most abundant cell population in ovarian follicles and the main source of E2 and PG in the ovary [39]. Since ALLO alters follicle maturation process, this neurosteroid might interfere with E2 secretion. The decline in E2 serum levels found in our experiments reflects the dysfunctionality of follicles as endocrine glands induced by ALLO. In a regular estrous cycle, once ovulation has occurred, E2 serum concentration begins to increase to reach 40 pg/ml in PE [40]. However, our results showed that E2 serum levels were significantly reduced at the following PE (96 hs) after ALLO administration. This is not the first time that a correlation is established between follicular atresia and low E2 levels [41]. Although the activity of steroidogenic enzymes involved in E2 secretion was not affected, we assume that the low levels of E2 are related to ALLO effects on follicular atresia. In addition, the ovary is not the only sex steroid-producing gland; the adrenal gland of the rat secretes E2 at rates similar to the ovary and brain in minor concentrations, and the possible effect of ALLO on this gland and its secretion is still unknown [42]. It would be interesting to evaluate proliferation and apoptosis in granulosa and theca cells of cystic follicles, the expression of pro-apoptotic and anti-apoptotic regulatory proteins, the balance between them (i.e.: BAX/BCL2) and the expression of steroid and gonadotropin receptors.

We have previously reported that ALLO caused a decrease in LH serum concentration of E rats [22], critical stage of the cycle in which LH induces ovulation. In the present research, we found that ALLO also decreased LH serum levels in PE rats as well. It has been shown that exogenous ALLO inhibits GnRH and LH secretion in rats through GABA action [21, 22]. These effects might be an outcome of the failure of the E2 negative feedback on GnRH [21, 43]. These results provide strong evidence that ALLO alters the cross-talk between the ovary and the hypothalamus, and thus ALLO causes the hypothalamus-hypophysis-ovarian (HHO) axis to dysregulate.

On the other hand, our results showed that ALLO injection increased the number of cystic structures. The neuro-endocrine dysfunction of the HHO axis is closely associated with cystic ovarian disease pathogenesis (COD). As it is well known, cysts progression and persistence during the estrous cycle can lead to ovarian failure, and they are considered an abnormality in folliculogenesis [44]. Cystic ovarian follicles represent an ovarian disorder and an important cause of subfertility [30]. Although ALLO involvement in COD is still being investigated, it is well known that women with polycystic ovary have high levels of this PG metabolite. It is unknown whether elevated concentration and/or prolonged stimulation with ALLO in polycystic disease can cause the development of tolerance to it [45]. It has been shown that exposure of female rats to E2 induced a dysregulation of gonadal axis and a decreased brain and plasma ALLO [46]. Furthermore, GABA_A_ receptors are involved in the pathophysiology of polycystic ovary syndrome [47, 48]. ALLO is able to alter GABA_A_ receptor sensitivity [49]. Therefore, we conclude that the dysregulation of the HHO axis by ALLO injection contributes to the formation of cystic structures, probably by altering GABA_A_ receptor sensibility to ALLO. It would be interesting to evaluate the expression of GABA receptors in the theca and granulosa cells of cystic structures.

The effects we have found could not only be related to the action of ALLO on GABA receptor but also they could be due to the action of ALLO over other ligands. ALLO affects brain function through actions at their cognate receptors (E2 receptor, PG receptor), or by modulating receptors whose primary transmitter is not a steroid (e.g. the GABA_A_ receptor). Furthermore, previous works show that GABA_A_ receptor is present not only in the CNS but also in the ovaries [50, 51]. Therefore, we assume that ALLO could exert its effects either at the brain or in the periphery (ovaries or adrenal gland). GABA_A_ has different functions with respect to specific cell types in peripheral tissues as ovary [51]. GABA_A_ receptor complex has been considered as the primary target of ALLO and majority of its inhibitory actions are mediated through GABA potentiation or direct activation of GABA currents [52]. However, GABA_A_ receptor are highly expressed in endocrine tissues, particularly in the ovaries: the ovary express a total of 14 or 15 receptor subunit subtypes [53]. The high GABA content of these tissues supports the significant physiological role of GABA and GABA_A_ receptors in ovaries. The results we present in the present article suggest that GABA receptors are involved in the regulation of follicular and luteal dynamics. We hypothesize that the action of ALLO might be mediated through GABA_A_ receptor in the ovaries. Further research is needed to evaluate the possible mechanism of action of ALLO and the signaling pathways involved in the ovaries. The specific site of ALLO action (central or peripheral) still remains unclear; however, we assume there might be more than one tissue that responds to ALLO effects.

The angiogenic process is critical for the *corpus luteum* development and regression [28]. PG serum concentration depends on the number and size of steroidogenic luteal cells (small and large), blood stream to the CL and on the ability of the steroidogenic tissue to synthesize PG. Mature CL receive most of blood ovarian irrigation and this could influence PG secretion [54]. Our results indicate that ALLO enhanced ovarian vascularization, increased ovarian 3β-HSD enzymatic activity in E (enzyme which converts pregnenolone in PG), and increased PG serum levels at D1. PCNA and von Willebrand staining pattern are similar, and they indicate that the cell type proliferating are endothelial cells. Moreover, PCNA staining in small luteal cells was increased in ALLO groups. It has been shown that PG interferes with luteal regression by a decrease in the number of apoptotic luteal cells and an increase in androstenedione levels [55]. Besides, it has been proved that PG maintains the functionality of CL through Notch pathway [45]. ALLO might be exerting a similar effect on CL in our experiments since PG levels and CL proliferation index are increased. In this work we found that ALLO increased proliferation and decreased apoptosis of luteal cells at all stages of the estrous cycle. Furthermore, we previously observed a decrease in the number of apoptotic *nuclei* in luteal cells from treated E rats [22]. Therefore, we could conclude that ALLO has a protective role on the function and survival of the CL. Further research is needed to distinguish CL cells types that undergo proliferation or apoptosis.

 McCarthy et al., 1995 [56] showed that changes in serum ALLO concentration play an important role in the normal facilitation of estrous behavior in the rat; ALLO provoke this effect by acting at least in part through the PG receptor. In previous work, we found that ALLO inhibited lordosis behavior in rats through GABA action [18]. Micevych & Sinchak, 2008 [43] showed that circuits involved in controlling the LH surge and sexual behaviors were thought to be influenced by E2 and PG synthesized in the ovary and perhaps in the adrenal. It is now apparent that E2 of ovarian origin regulates the synthesis of neuroprogesterone (neuro-PG), and it is the locally produced neuro-PG that is involved in the initiation of the LH surge and subsequent ovulation. Moreover, E2 induces the transcription of PG receptors while stimulating synthesis of neuro-PG. Although the complete signaling cascade has not been elucidated, many of the features have been characterized.

## Conclusion

The major findings of the present research article are that ALLO (1) interferes with follicular maturation affecting apoptosis/proliferation balance, (2) increases follicular atresia and cysts formation, (3) delays luteolysis by increasing serum PG and 3β-HSD enzymatic activity. ALLO has novel functions, which have not previously been identified in the rat ovary; thereby, these studies provide new insights about critical process for ovarian homeostasis (proliferation, apoptosis, angiogenesis and steroidogenesis). The effects of ALLO on these processes contribute to dysregulate the hypothalamus-hypophysis axis, and this results in inhibition of ovulation by neurosteroid action. On the basis of our discussion, the effects of ALLO strongly depend on the ovarian hormonal milieu that changes along the different stages of the estrous cycle. In order to comprehend the molecular mechanisms of ALLO action, a greater understanding of the role of hormonal milieu is strongly necessary.

## Abbreviations

20α-HSD: 20α hydroxysteroid dehydrogenase
3α-HSOR: 3α-hydroxysteroid oxidoreductase
3β-HSD: 3β-hydroxysteroid dehydrogenase
AF: Antral follicle
ALLO: Allopregnanolone
ATF: Atretic follicles
CASP3: Caspase 3
CL: *Corpora lutea*
CNS: Central nervous system
D1: Diestrous 1
D2: Diestrous 2
E: Estrous
E2: 17β-estradiol
EAF: Early antral follicles
GF: Graafian follicles
LH: Luteinizing hormone
LUF: Luteinized unruptured follicles
MBH: Medial basal hypothalamus
neuroPG: Neuroprogesterone
PAF: Preantral follicles
PCNA: Proliferating cell nuclear antigen
PE: Proestrous
PF: Primary follicles
PG: Progesterone
PRL: Prolactin
